# Serum Neuroglobin as a Potential Prognostic Biomarker for Cognitive Impairment After Intracerebral Hemorrhage

**DOI:** 10.3389/fneur.2022.885323

**Published:** 2022-04-07

**Authors:** Yu Gao, Bo Wang, Ye Miao, Yu Han

**Affiliations:** ^1^Department of Cardiology, The First Affiliated Hospital of Jinzhou Medical University, Jinzhou, China; ^2^Department of Neurosurgery, The First Affiliated Hospital of Jinzhou Medical University, Jinzhou, China; ^3^Department of Emergency, The First Affiliated Hospital of Jinzhou Medical University, Jinzhou, China

**Keywords:** intracranial hemorrhage, neuroglobin, post-stroke cognitive impairment, prognosis, biomarker

## Abstract

**Objective:**

Stroke is closely related to dementia, but there are few prospective studies on cognitive decline after stroke in patients with cerebral hemorrhage. Neuroglobin is an oxygen-binding protein mainly expressed in brain neurons. The aim of our current study was to determine whether neuroglobin could serve as a biomarker for cognitive prognosis in patients with intracerebral hemorrhage (ICH).

**Methods:**

Three hundred and sixteen patients with ICH were consecutively enrolled in a prospective study. Baseline data such as age and gender of ICH patients on admission were recorded. Serum neuroglobin concentrations were determined by enzyme-linked immunosorbent assay (ELISA). All ICH patients 3 months after onset were divided into post-stroke cognitive impairment group (PSCI) and non-PSCI group according to MoCA assessment results.

**Results:**

The PSCI and Non-PSCI groups had serum neuroglobin concentrations of (4.7 ± 0.9) and (7.5 ± 1.1) ng/ml, respectively, with a statistically significant difference between the two groups (*p* < 0.05). Age, gender, LDL, FBG, SBP, DBP, NHISS, and Hematoma volume were found to be adversely connected with MoCA (*p* < 0.05), while education, HDL, and serum neuroglobin were found to be positively correlated with MoCA (*p* < 0.05). After controlling for baseline data, regression analysis revealed that serum neuroglobin was remained an efficient biomarker for predicting cognitive performance in individuals with ICH (*p* < 0.05). The diagnostic accuracy of blood neuroglobin concentration for PSCI in ICH patients was 72.6%, the sensitivity was 67.4%, and the specificity was 75.5%, according to receiver operating characteristic (ROC) curve analysis.

**Conclusions:**

Serum neuroglobin may serve as a potential biomarker to predict cognitive decline after ICH.

## Introduction

Intracranial hemorrhage (ICH) is defined as any type of intracranial hemorrhage that kills neurons and pressures surrounding brain tissue, causing neurological impairments (1, 2). ICH accounts for 10–20% of all strokes and is the second most common subtype of stroke, with significant impairment or death as a result (3, 4). The prevalence of ICH varies greatly between countries and ethnic groups, with ICH in low- and middle-income countries being twice as common as in high-income countries (5). The incidence of ICH is 8–15% in Western countries such as the United States, United Kingdom and Australia, 18–24% in Japan and South Korea, and 14.9% in China (6–8). The average cost of initial hospitalization for ICH survivors was $28,360 and the first year after discharge was $16,035, with 8% requiring repeat hospitalizations and 41% unable to take care of themselves (9). The high morbidity, high cost, and disability rate of ICH make it an important factor restricting social and economic development, and the search for the potential etiology and biological targets of ICH has become an urgent need.

The globin isoform neuroglobin is found in both the central and peripheral neural systems (10). Thorsten Burmester et al. were the first to find it in 2000 (11). Its primary role is in maintaining cellular oxygen homeostasis and scavenging reactive oxygen species and nitrogen. It also improves brain tissue's oxygen supply capacity and protects it from hypoxic or ischemia damage, potentially lowering ischemic hypoxic brain injury (12, 13).

Cognitive impairment appears to be a common sequelae of (ICH) survivors, but effective prevention and treatment methods are still lacking (14, 15). Neuroglobin, as a potential neuroprotective protein discovered in recent years, is endowed with relatively broad application prospects (16). The goal of our research was to see if neuroglobin may be used as a biomarker for cognitive impairment following ICH.

## Methods

### Study Population

**Figure 1:**
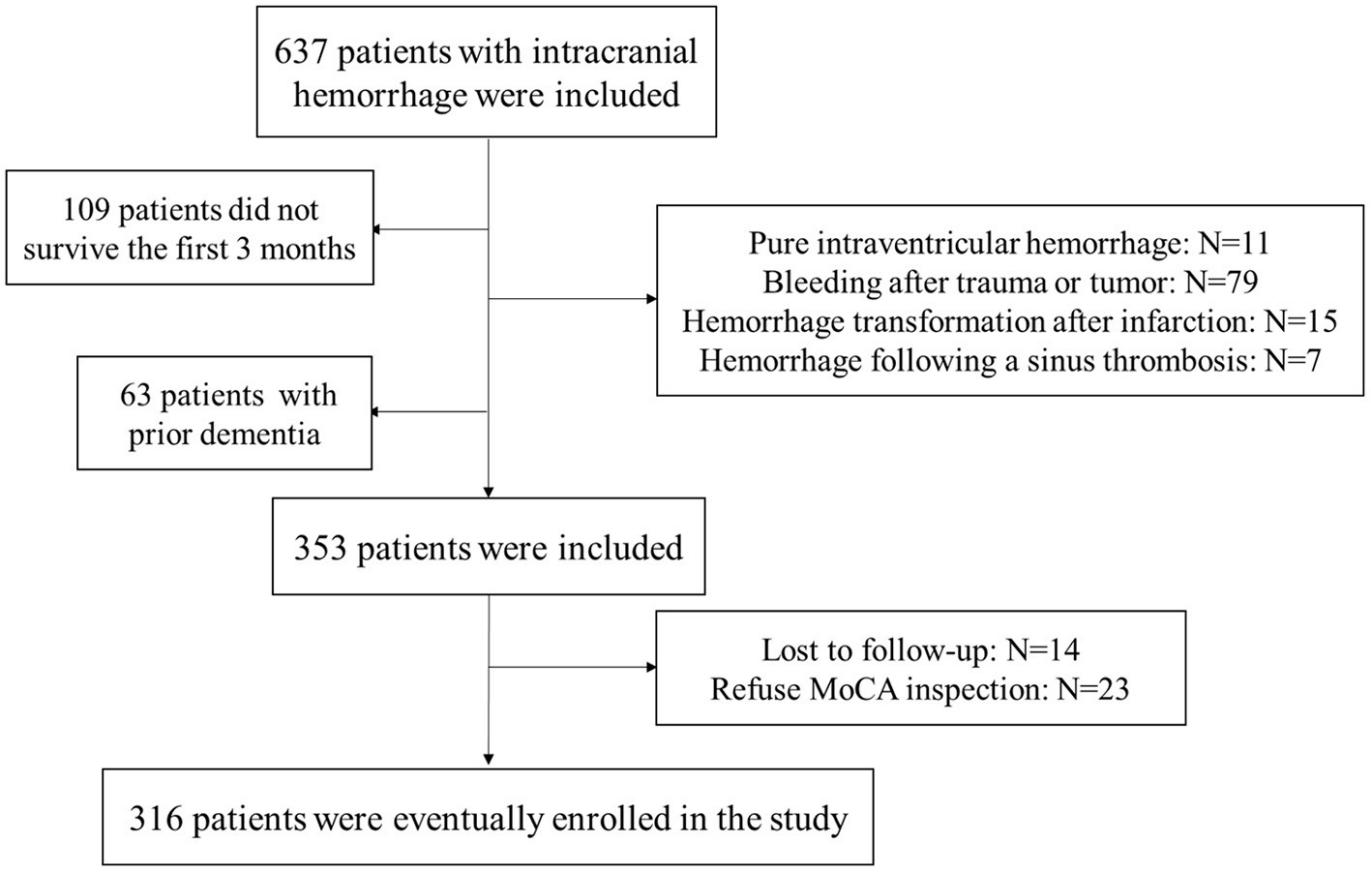
Flow chart of the study. MoCA, Montreal Cognitive Assessment.

Six hundred and thirty-seven patients with ICH were prospectively enrolled for screening and follow-up. The inclusion criteria for patients with cerebral hemorrhage were: within 24 h of onset, and confirmed ICH by cranial CT. The exclusion criteria for ICH patients were: pure intraventricular hemorrhage (*N* = 11), bleeding after trauma or tumor (*N* = 79), bleeding after trauma or tumor (*N* = 79), hemorrhage following a sinus thrombosis (*N* = 7), not survive the first 3 months (*N* = 109), prior dementia (*N* = 63), lost to follow-up (*N* = 14) and refuse MoCA inspection (*N* = 23). According to the inclusion and exclusion criteria, a total of 316 ICH patients were eventually included in the study. Figure 1 depicts a detailed flowchart. Our study was approved by the local medical ethics committee, and the application of clinical data was with the permission of all participants or their families.

### Baseline Data Collection

After enrolment, all ICH patients' baseline data were obtained. Age, gender, years of schooling, low density lipoprotein (LDL), high density lipoprotein (HDL), fasting plasma glucose (FBG), systolic blood pressure (SBP), diastolic blood pressure (DBP), National Institutes of Health Stroke Scale (NHISS), and Hematoma volumes were among the information collected. Statistical analysis of all baseline data was performed by professional personnel.

### Serum Neuroglobin Concentration Determination

Within 24 h after the commencement of ICH, nurses drew venous blood from the patients. The venous blood was taken and left to sit at room temperature for 20 min before being centrifuged at high speed to extract serum. Serum was aliquoted and stored in a −80°C freezer. Purchased ELISA reagents from MyBioSource (San Diego, CA, USA) were used to detect serum neuroglobin concentrations. The specific experimental steps of ELISA refer to previous reports and product instructions (17).

### MoCA Scale Evaluation

All ICH patients underwent cognitive function assessment 3 months after onset. The Montreal Cognitive Assessment (MoCA) is a worldwide cognitive screening tool founded in 1996 by Ziad Nasreddine in Montreal, Quebec. MoCA has a total score of 30 points, and the test content includes short-term memory, executive performance, attention, concentration, etc. (18). Compared with the well-known Mini Mental State Examination (MMSE), MoCA is a promising tool for the detection of mild cognitive impairment (MCI) and early Alzheimer's disease. The test is also being used in hospitals to decide whether to allow a patient to live alone or with a home assistant.

### Statistical Analysis

Categorical variables are expressed as numbers, while continuous variables are expressed as mean and standard deviation (percent). The *t*-test and the chi-square test were used to compare continuous and categorical variables, respectively. The underlying etiologies affecting cognitive outcomes in patients with ICH were investigated using correlation and regression analysis. The predictive efficacy of serum neuroglobin levels for cognitive deterioration following ICH was assessed using a receiver operating characteristic (ROC) curve analysis. This study employed SPSS 22.0 (SPSS Inc., Chicago, IL, USA) for analysis, with *p*-values < 0.05 considered statistically significant.

## Results

### Baseline Data

A total of 316 patients with cerebral hemorrhage were included in the study after screening. According to the MoCA score 3 months after ICH, ICH patients were separated into two groups: PSCI (MoCA 26) and non-PSCI (MoCA 26). Table 1 summarizes the baseline data of all subjects. The statistical results revealed that there was no significant difference between the two groups in terms of age, gender, years of education, LDL, HDL, FBG, SBP, DBP, NHISS, and hematoma volume (*p* > 0.05).

**Table 1:** Baseline characteristics of ICH patients in the PSCI and non-PSCI groups.

	**Non-PSCI group** *****N*** = 124**	**PSCI group** *****N*** = 192**	***P*** **-value**
Age, years	65.3 ± 8.2	65.7 ± 7.8	0.663
Gender, male/female	75/49	120/72	0.719
Education, years	10.1 ± 2.5	10.3 ± 2.1	0.444
LDL, mmol/L	2.37 ± 0.18	2.35 ± 0.14	0.269
HDL, mmol/L	1.12 ± 0.13	1.10 ± 0.09	0.107
FBG, mmol/L	6.14 ± 0.81	6.16 ± 0.87	0.838
SBP, mmHg	137.6 ± 11.2	137.1 ± 10.3	0.684
DBP, mmHg	84.5 ± 7.6	84.8 ± 7.0	0.719
NHISS, points	12.8 ± 2.4	13.2 ± 2.6	0.170
Hematoma (ml)	14.0 ± 6.7	14.4 ± 6.5	0.598
MoCA, points	27.1 ± 0.8	24.3 ± 1.2	<0.001
Neuroglobin, ng/ml	7.5 ± 1.1	4.7 ± 0.9	<0.001

*LDL, low density lipoprotein; HDL, high density lipoprotein; FBG, fasting plasma glucose; SBP, systolic blood pressure; DBP, diastolic blood pressure; NHISS, National Institutes of Health Stroke Scale; MoCA, Montreal Cognitive Assessment*.

**Figure 2:**
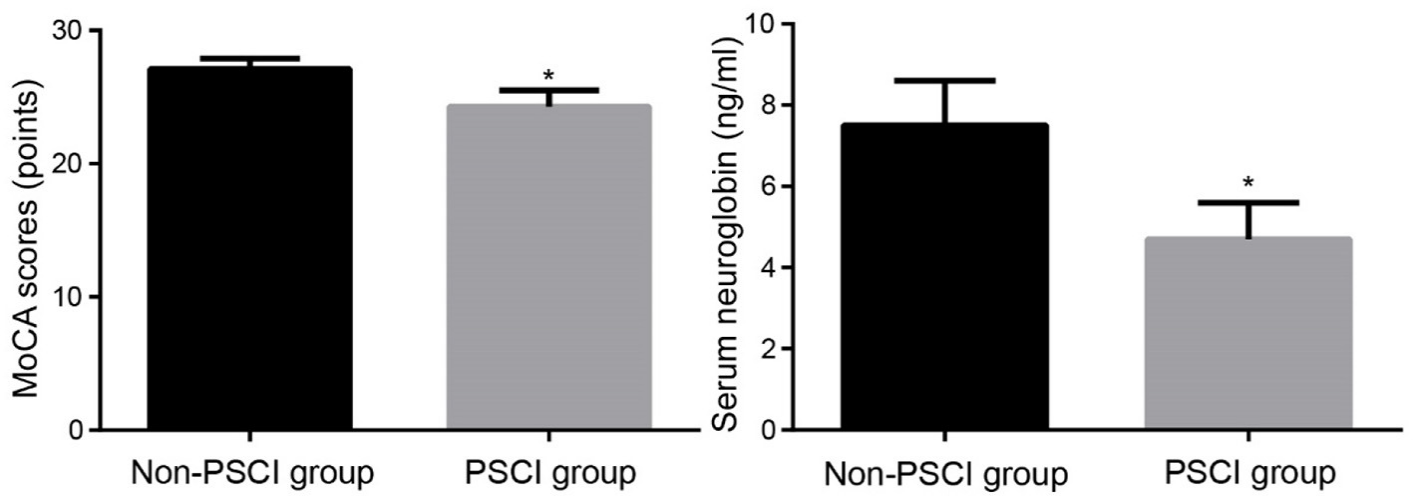
Comparison of MoCA scores and serum neuroglobin concentrations between groups. MoCA, Montreal Cognitive Assessment; PSCI, post-stroke cognitive impairment. Compared to Non-PSCI group, ^*^P < 0.05.

The MoCA scores of the PSCI and non-PSCI groups, on the other hand, were (24.3 ± 1.2) and (27.1 ± 0.8), respectively, with a statistically significant difference between the two groups (*p* < 0.001). Similarly, the PSCI and non-PSCI groups had blood neuroglobin concentrations of (4.7 ± 0.9) ng/ml and (7.5 ± 1.1) ng/ml, respectively, with a statistically significant difference between the two groups (*p* < 0.001). Figure 2 shows a comparison of MoCA score and serum neuroglobin concentration.

### Correlation Analysis

Correlation analysis showed that age, gender, LDL, FBG, SBP, DBP, NHISS and hematoma volume were negatively correlated with MoCA (*p* < 0.05), while education, HDL and serum neuroglobin were positively correlated with MoCA (*p* < 0.05). The specific correlation coefficients are shown in Table 2.

**Table 2:** Correlation analysis of MoCA scores with baseline characteristics.

	**Spearman's correlation coefficient**	***P*** **-values**
Age, years	−0.142	<0.001
Gender, male/female	−0.205	0.036
Education, years	0.178	<0.001
LDL, mmol/L	−0.113	<0.001
HDL, mmol/L	0.164	<0.001
FBG, mmol/L	−0.193	<0.001
SBP, mmHg	−0.237	0.142
DBP, mmHg	−0.221	0.097
NHISS, points	−0.186	<0.001
Hematoma (ml)	−0.259	0.028
Neuroglobin, ng/ml	0.307	<0.001

*MoCA, Montreal Cognitive Assessment; LDL, low density lipoprotein; HDL, high density lipoprotein; FBG, fasting plasma glucose; SBP, systolic blood pressure; DBP, diastolic blood pressure; NHISS, National Institutes of Health Stroke Scale*.

### Regression Analysis

The results of the regression analysis are shown in Table 3. In Model 1, after adjusting for sex, age, and years of education, serum neuroglobin concentration was an independent risk predictor for PSCI (β = 0.356, *p* < 0.001). In Model 2, after further adjustment for LDL, HDL, FBG, SBP, and DBP, serum neuroglobin concentration was also an independent risk predictor for PSCI after ICH (β = 0.329, *p* = 0.017). In Model 2, after further adjustment for NHISS scores and Hematoma volumes, serum neuroglobin concentration was also an independent risk predictor for PSCI after ICH (β = 0.311, *p* = 0.038).

**Table 3:** Regression analysis of serum neuroglobin levels and MoCA scores.

	**Regression coefficient**	***P*** **-values**
Model 1	0.356	<0.001
Model 2	0.329	0.017
Model 3	0.311	0.038

*Model 1: adjusted for age, gender and education; Model 2: further adjusted for LDL, HDL, FBG, SBP and DBP; Model 3: further adjusted for NHISS scores and Hematoma volumes*.

*MoCA, Montreal Cognitive Assessment; LDL, low density lipoprotein; HDL, high density lipoprotein; FBG, fasting plasma glucose; SBP, systolic blood pressure; DBP, diastolic blood pressure; NHISS, National Institutes of Health Stroke Scale*.

### ROC Analysis

**Figure 3:**
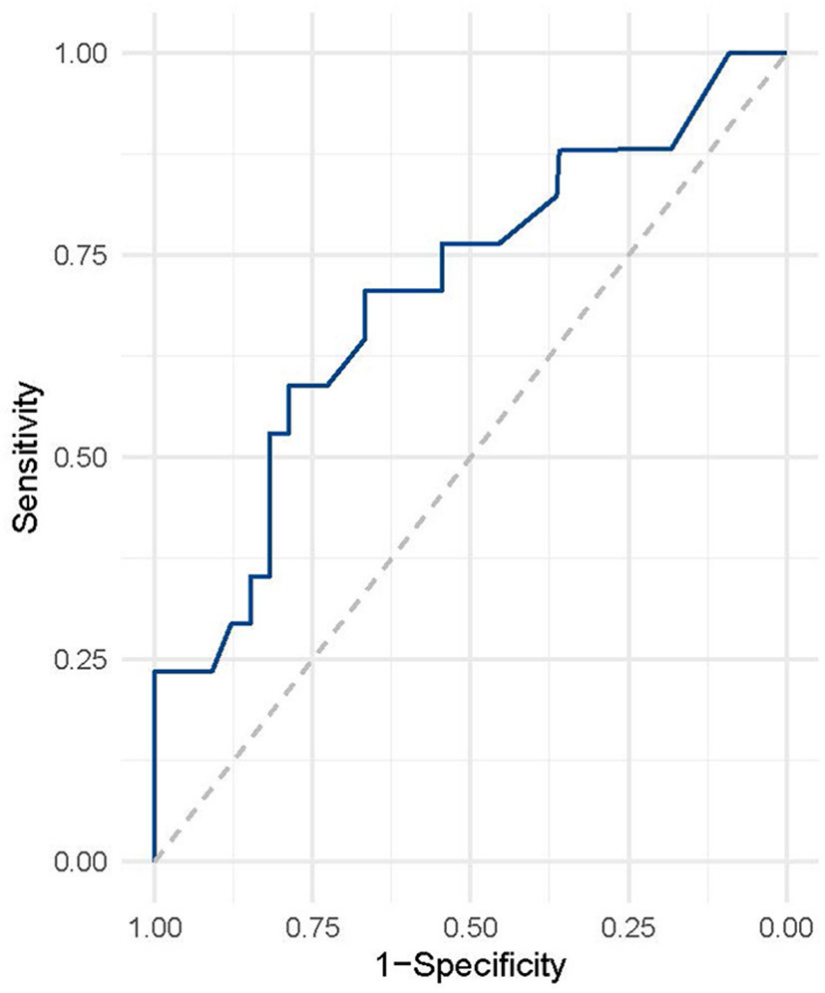
ROC analysis of serum neuroglobin for predicting PSCI after ICH. ROC, receiver operating characteristic; ICH, intracranial hemorrhage.

We used ROC analysis to determine the predictive value of serum neuroglobin concentration for PSCI following ICH, as shown in Figure 3. In the diagnosis of PSCI following ICH, serum neuroglobin concentration had a sensitivity, specificity, and accuracy of 67.4, 75.5, and 72.6%, respectively. For the diagnosis of poor prognosis in ICH, the threshold value for serum neuroglobin concentration was 5.9 ng/mL.

## Discussions

We investigated the value of serum neuroglobin in predicting cognitive impairment after ICH. Our study found that serum neuroglobin concentrations were significantly lower in PSCI patients than in non-PSCI patients after ICH. Correlation regression analysis found that serum neuroglobin was an independent predictor of PSCI after ICH. Our further ROC analysis found that neuroglobin has high sensitivity and specificity in predicting PSCI after ICH, suggesting that it has a high value as a predictor of PSCI after ICH.

Neuroglobin is a monomeric hexametric heme protein with a molecular weight of 17 kda, belonging to the globin family, which is mainly expressed in neurons of the central and peripheral nervous systems (19, 20). Neuroglobin is a heme protein that is highly conserved during evolution, with more than 90% genetic sequence identity between humans and mice (21, 22). Neuroglobin is widely distributed in the human body, including the hippocampus, diencephalon, cerebral cortex, cerebellum, organs with endocrine functions, and retinal cells (23). Neuroglobin has a common 8 α-helix structure, and its main physiological function is to bind and transport oxygen, scavenge and detoxify reactive oxygen species (24). The heme prosthetic group of neuroglobin that transports oxygen is located between the fifth and sixth helices. Neuroglobin is mainly expressed intracellularly, and it cannot pass through normal cell membranes and blood-brain barrier, which limits its application (25–27).

Neuroglobin has been found to have neuroprotective effects in recent years. The research of Professor Jin Kunlin's team showed that the neuroglobin level in the autopsy brain tissue of Alzheimer's disease patients increased compared with the normal control group (28). Italian scholars have found that the causative gene of Huntington's disease can damage the 17β-estradiol/neuroglobin signaling pathway, thereby affecting neuronal survival, suggesting that neuroglobin is involved in the pathogenic mechanism of Huntington's disease (29). In addition, the expression of neuroglobin present in the substantia nigra and striatum was upregulated in a mouse model of Parkinson's disease, suggesting that neuroglobin may be involved in the mechanism of its pathogenesis (30, 31). The above studies suggest that neuroglobin has a neuroprotective role in neurodegeneration. Another study showed that neuroglobin could be detected in multiple regions after cerebral infarction, and its expression was significantly increased in the ischemic penumbra (32). Based on the neuroprotective effect of neuroglobin, scientists developed a hyaluronic acid nanoparticle delivery system that can pass through the BBB (33), breaking through the application dilemma of neuroglobin to a certain extent.

The role of neuroglobin in hemorrhagic stroke has also received increasing attention. Cai et al.'s study showed that serum neuroglobin concentration on the second day after subarachnoid hemorrhage was closely related to poor prognosis, suggesting that it may be a potential biological target for predicting poor prognosis of subarachnoid hemorrhage (34). Another study showed that neuroglobin expression was significantly increased in the surrounding neurons of arteriovenous malformation and ICH patients and in the perihematoma area near cerebral hemorrhage, which may be involved in neuroprotection after brain injury (35). A recent study showed that early serum neuroglobin concentration predicts delayed cerebral ischemia in patients with aneurysmal subarachnoid hemorrhage (36). However, the role of neuroglobin in ICH remains poorly understood.

Our study is the first to demonstrate that serum neuroglobin is associated with PSCI after ICH. However, our study also has some limitations. First, we are a single-center study and include no normal controls; second, our main research object is the northern Han population in China, and the findings may not be applicable to other regions and ethnic groups; third, there is a lack of *in vitro* and *in vivo* studies on the neuroprotective mechanism of neuroglobin.

## Conclusions

Serum neuroglobin concentration may be a potential biomarker for predicting PSCI after ICH. The correlation between serum neuroglobin and PSCI after ICH needs to be further studied, and the research results may provide new prospects for the prevention and treatment of PSCI after ICH.

## Data Availability Statement

The raw data supporting the conclusions of this article will be made available by the authors, without undue reservation.

## Ethics Statement

The studies involving human participants were reviewed and approved by Ethics Committee of Jinzhou Medical University. The patients/participants provided their written informed consent to participate in this study.

## Author Contributions

YH designed this research and wrote the manuscript. YG, BW, and YM participated in experimental process and statistical analysis. All authors approved the submitted version.

## Conflict of Interest

The authors declare that the research was conducted in the absence of any commercial or financial relationships that could be construed as a potential conflict of interest.

## Publisher's Note

All claims expressed in this article are solely those of the authors and do not necessarily represent those of their affiliated organizations, or those of the publisher, the editors and the reviewers. Any product that may be evaluated in this article, or claim that may be made by its manufacturer, is not guaranteed or endorsed by the publisher.
